# Molecular characterization of *4*-*Coumarate:* *CoA Ligase* gene family in orchids: an *in silico* study

**DOI:** 10.3389/fpls.2026.1895523

**Published:** 2026-07-23

**Authors:** Arshpreet Kaur, Sandip V. Pawar, Jaspreet K. Sembi

**Affiliations:** 1 Department of Botany, Panjab University, Chandigarh, India; 2 Zakir Husain Delhi College, University of Delhi, New Delhi, India; 3 University Institute of Pharmaceutical Sciences, Panjab University, Chandigarh, India

**Keywords:** 4-Coumarate: CoA Ligase (4CL), orchids, *p*-coumaric acid, phenylpropanoid pathway, secondary metabolites

## Abstract

The 4-Coumarate: CoA Ligase (4CL) is a crucial enzyme in the phenylpropanoid pathway, which leads to the biosynthesis of a diverse array of secondary metabolites. The present research identified 29 *4CL* genes encoding this enzyme in eight orchid species (*Apostasia shenzhenica*, *Dendrobium catenatum*, *Phalaenopsis aphrodite*, *Phalaenopsis bellina*, *Phalaenopsis equestris*, *Phalaenopsis modesta*, *Phalaenopsis lueddemanniana* and *Phalaenopsis schilleriana*). Domain and motif analyses revealed the relatively conserved nature of the identified proteins, and all were localized to the peroxisome. Phylogenetic analysis showed that they clustered into three different groups. A total of 54 types of *cis*-acting regulatory elements across four categories were found in the promoters of these genes, indicating their functional diversity. Varied expression profiles were observed for the genes in vegetative and reproductive tissues of the orchids under study, with 11 genes showing high expression in the root, underscoring their role in plant growth and development. To conclude, this research lays the groundwork for future studies involving the functional characterization of *4CL* genes and their correlation with the related phenylpropanoid synthesis.

## Introduction

Orchids belong to the family Orchidaceae, which is one of the largest and most advanced groups of flowering plants (Govaerts et al., 2026). Orchids have an astonishing therapeutic potential due to the presence of phytochemicals that can be extracted from different parts of the plant. Several classes of phytoconstituents, such as alkaloids, phenolics, terpenes, etc., have been isolated from therapeutically important orchids (Ghai et al., 2021). Amongst these diverse phytoconstituents, phenolics are a vast group of compounds consisting of one or more aromatic rings with one or more hydroxyl groups and synthesized through the phenylpropanoid pathway. Thus, this metabolic pathway leads to the synthesis of lignins, flavonoids, coumarins, suberins, and bibenzyls, etc., that possess therapeutic potential and also provide protection to the plants against various environmental stress (biotic and abiotic) conditions (Vogt, 2010). Recent studies have also highlighted the role of phenylpropanoid pathway genes in stress tolerance. For instance, in *Zea mays*, *flavanone 3-hydroxylase (F3H)* gene (*ZmF3H6*) enhanced salt stress tolerance by the overaccumulation of flavonols (Xiao et al., 2026).

The phenylpropanoid pathway is regulated by several key players, including phenylalanine ammonia lyase (PAL), which catalyzes the first step of deamination of phenylalanine, resulting in the production of *trans*-cinnamate (Vogt, 2010), which is further transformed into *p*-coumaroyl CoA through the action of cinnamate 4-hydroxylase (C4H) and 4-Coumarate: CoA Ligase (4CL). The 4-Coumarate: CoA Ligase (4CL) enzyme (EC 6.2.1.12) was first discovered in *Petroselinum crispum* (Douglas et al., 1987) to catalyze the transformation of *p*-coumaric acid into *p*-coumaroyl CoA (Ehlting et al., 1999). It plays a crucial role in directing carbon flux from the core phenylpropanoid pathway into the synthesis of various phenylpropanoid-derived compounds (Ehlting et al., 1999). Further, the products formed by the action of 4CL enzyme act as precursors for the synthesis of varied metabolites, playing a role in tolerance towards abiotic and biotic stresses, lignification and UV protection (Lavhale et al., 2018).

The 4CL enzymes have been classified into three clusters: class I, class II, and class III, based on evolutionary analysis (Ehlting et al., 1999; Costa et al., 2003). Sequence alignments have consistently revealed the presence of conserved peptide motifs in all available 4-coumarate: CoA ligase (4CL) amino acid sequences (Becker-André et al., 1991). Among these motifs, the Box I motif, represented by SSGTTGLPKGV, exhibits near-absolute conservation in 4CLs, and similar motifs are prevalent in diverse enzymes, including luciferases, acetyl-CoA synthetases, long-chain fatty acyl-CoA synthetases, and peptide synthetases. Furthermore, the Box II motif, GEICIRG, is universally conserved across all 4CLs (Becker-André et al., 1991; Uhlmann and Ebel, 1993; Hu et al., 1998). The central cysteine residue within this motif has been proposed to play a direct role in catalytic processes (Stuible et al., 2000). This highlights the significance of these motifs in the functional and structural aspects of 4CLs.

The number of members included in the *4CL* gene family also varies amongst different species of the plant kingdom like *Arabidopsis thaliana* (4; Li et al., 2015), *Boehmeria nivea* (3; Tang et al., 2018), *Citrus sinensis* (3; Zhou et al., 2022), *Glycine max* (3; Lindermayr et al., 2002), *Oryza sativa* (5; Gui et al., 2011), *Panicum virgatum* (2; Xu et al., 2011), *Physcomitrella patens* (4; Silber et al., 2008), *Piper nigrum* (3; Jin et al., 2020), *Populus tomentosa* (5; Rao et al., 2015), *Punica granatum* (12; Wang et al., 2022), *Rubus idaeus* (3; Kumar and Ellis, 2003), and *Salvia miltiorrhiza* (2; Jin et al., 2012). To date, the maximum number of *4CL* genes has been reported in *Malus domestica* (69; Ma et al., 2023) (**Table 1**). These variations in the number of genes in the *4CL* gene family in different plants suggest that different *4CL* genes may lead to the biosynthesis of different kinds of phenylpropanoids.

**Table 1:** Numerical strength of the *4CL* gene family across various plant species.

Name of the plant	Name of the family	Number of members	Reference
*Arabidopsis thaliana*	Brassicaceae	4	Li et al., 2015
*Beta vulgaris*	Amaranthaceae	27	Lan et al., 2026
*Bletilla striata*	Orchidaceae	21	Huang et al., 2023
*Boehmeria nivea*	Urticaceae	3	Tang et al., 2018
*Brassica carinata*	Brassicaceae	10	Zhang M. et al., 2025
*Brassica juncea*	Brassicaceae	13	Zhang M. et al., 2025
*Brassica napus*	Brassicaceae	12	Zhang M. et al., 2025
*Brassica nigra*	Brassicaceae	6	Zhang M. et al., 2025
*Brassica oleracea*	Brassicaceae	5	Zhang M. et al., 2025
*Brassica rapa*	Brassicaceae	7	Zhang M. et al., 2025
*Camellia sinensis ‘Huangdan’*	Theaceae	8	Ren et al., 2023
*Camellia sinensis ‘Tie-guanyin’*	Theaceae	9	Ren et al., 2023
*Citrus sinensis*	Rutaceae	3	Zhou et al., 2022
*Gastrodia elata*	Orchidaceae	14	Sha et al., 2025
*Glycine max*	Fabaceae	20	Yang et al., 2024
*Hippophae rhamnoides*	Elaeagnaceae	7	Song et al., 2023
*Juglans regia*	Juglandaceae	36	Ma et al., 2024
*Juglans mandshurica*	Juglandaceae	31	Ma et al., 2024
*Lithospermum erythrorhizon*	Boraginaceae	8	Nakanishi et al., 2024
*Malus domestica*	Rosaceae	69	Ma et al., 2023
*Manihot esculenta*	Euphorbiaceae	50	Ran et al., 2024
*Marchantia paleacea*	Marchantiaceae	4	Gao et al., 2024
*Miscanthus lutarioriparius*	Poaceae	20	Huang et al., 2026
*Musa acuminata*	Musaceae	20	Wang et al., 2024
*Nicotiana tabacum*	Solanaceae	24	Ma et al., 2026
*Oryza sativa*	Poaceae	5	Gui et al., 2011
*Panicum virgatum*	Poaceae	2	Xu et al., 2011
*Physcomitrella patens*	Funariaceae	4	Silber et al., 2008
*Piper nigrum*	Piperaceae	3	Jin et al., 2020
*Populus tomentosa*	Salicaceae	5	Rao et al., 2015
*Punica granatum*	Lythraceae	12	Wang et al., 2022
*Pyrus bretschneideri*	Rosaceae	18	Zhang et al., 2024
*Rubus idaeus*	Rosaceae	3	Kumar and Ellis, 2003
*Salvia miltiorrhiza*	Lamiaceae	10	Fu et al., 2025
*Solanum tuberosum*	Solanaceae	10	Nie et al., 2023
*Triticum aestivum*	Poaceae	40	Gholizadeh et al., 2025
*Zea mays*	Poaceae	32	Zhang Z et al., 2025a

Researchers have extensively investigated the expression patterns of *4CL* genes in multiple plant species across various tissues and developmental stages (Hu et al., 1998; Ehlting et al., 1999; Kumar and Ellis, 2003; Di et al., 2012; Jin et al., 2012; Tang et al., 2018; Jin et al., 2020). Furthermore, the response of *4CL* genes to different stimuli has been explored. Investigations have focused on their expression changes in relation to elicitors/phytohormones, as demonstrated by the work of Awasthi et al. (2019) in *Coleus forskohlii*. Additionally, the impact of abiotic stress on the expression of *4CL* genes has been studied by Sun et al. (2020) in *Gossypium hirsutum* and Zhong et al. (2022) in *Eucommia ulmoides* and Ma et al. (2026) in *Nicotiana tabacum.* The role of *4CL* genes in antifungal response has also been reported in *Gastrodia elata* (Sha et al., 2025). Another factor that has been investigated is the influence of UV exposure on the expression patterns of *4CL* genes in *Isatis indigotica*, as observed in the study conducted by Di et al. (2012). By examining their expression patterns across diverse tissues, developmental stages, and in response to various environmental triggers, scientists have gained a better understanding of the roles and regulatory mechanisms of *4CL* genes in plant growth, development, and stress responses. Additionally, overexpression of specific *4CL* genes leading to increased metabolite synthesis or tolerance to stress conditions has been reported in certain plants, such as *Fraxinus mandshurica*, wherein overexpression of the *Fm4CL2* gene conferred resistance to osmotic and drought stress (Chen et al., 2020). Thus, these studies have improved our understanding of the genetic diversity, functional roles, and potential applications of *4CL* genes in different plant species, highlighting their importance in plant biology.

Thus, keeping both the significance of *4CL* genes and the therapeutic importance of orchids in view, the present study on the identification and characterization of *4CL* genes in eight selected orchid species (*Apostasia shenzhenica*, *Dendrobium catenatum*, *Phalaenopsis aphrodite*, *Phalaenopsis bellina*, *Phalaenopsis equestris*, *Phalaenopsis modesta*, *Phalaenopsis lueddemanniana*, and *Phalaenopsis schilleriana*) was conducted. Further, this study acts as a reference template for conducting similar studies on these genes in various plants to delve into the plausible functions of the *4CL* genes.

## Materials and methods

### Identification of 4CL genes in selected orchid species

For the current study, genome data of *A. shenzhenica* [Accession: PRJNA310678], *D. catenatum* [Accession: PRJNA262478] and *P. equestris* [Accession: PRJNA192198, PRJNA382149] was taken from the National Center for Biotechnology Information (NCBI) (https://www.ncbi.nlm.nih.gov/). 4CLs (Gui et al., 2011; Li et al., 2015) of *Arabidopsis thaliana* and *Oryza sativa* were retrieved from NCBI and Phytozome using their accession numbers (**Supplementary Table 1**). These sequences were used as a query to execute a BLASTp search against the protein sequences of *A. shenzhenica, D. catenatum* and *P. equestris* using the NCBI Protein BLAST web server against the ClusteredNR database. The search was conducted using the default BLASTp settings, including the BLOSUM62 substitution matrix, conditional compositional score matrix adjustment and default gap costs. The hits showing an E-value of zero and sequence identity greater than 50% were subjected to further validation. The 4CL protein sequences of *A. thaliana* and *O. sativa* (**Supplementary Table 1**) were used to conduct a tBLASTn search for the identification of these specific proteins from the transcriptomes of *P. aphrodite*, *P. bellina, P. lueddemanniana*, *P. modesta* and *P. schilleriana* in the Orchidstra 2.0 database (Chao et al., 2017; http://orchidstra2.abrc.sinica.edu.tw/orchidstra2/index.php).

### Domain analysis and multiple sequence alignment

Conserved domain search was employed using the SMART (Letunic et al., 2015; http://smart.embl-heidelberg.de/) tool to confirm the presence of the AMP-binding (PF00501) and AMP-binding_C (PF13193) domains, which are conserved for 4CL proteins. After the recognition of the domain, all the protein sequences were subjected to Expasy-PROSITE (Sigrist et al., 2012; https://prosite.expasy.org/) for the construction of domain architecture. The conserved region of 4CL proteins was located and marked by using the MultAlin server (Corpet, 1988; http://multalin.toulouse.inra.fr/multalin/) by sequence alignment of the identified 4CL proteins along with the 4CLs of *Arabidopsis thaliana* and *Oryza sativa.*

### Conserved motif analysis

Conserved motifs and their positional distribution within the identified 4CL proteins from *A. shenzhenica*, *D. catenatum*, *P. aphrodite*, *P. bellina*, *P. equestris*, *P. lueddemanniana*, *P. modesta* and *P. schilleriana* along with their counterparts from *Arabidopsis thaliana* and *Oryza sativa*, were predicted using the Multiple Expectation Maximization for Motif Elicitation (MEME) Suite (version 5.5.5) (Bailey et al., 2015; https://meme-suite.org/meme/). The analysis permitted any number of motif repetitions, with the maximum number of motifs set to 10. The minimum and maximum motif widths were defined as 6 and 50 amino acids, respectively, while all other parameters were maintained at their default settings.

### Physicochemical characterization and sub-cellular localization

Protein physicochemical properties, including sequence length (total amino acids), molecular weight, isoelectric point (pI), instability index, aliphatic index, and GRAVY (grand average of hydropathicity) values, were calculated using ProtParam (Gasteiger et al., 2005; https://web.expasy.org/protparam/). Subcellular localization of the proteins was predicted using the Plant-mPLoc server (Chou and Shen, 2010; http://www.csbio.sjtu.edu.cn/bioinf/plant-multi/).

### Secondary and tertiary structure prediction

The secondary structural features of the proteins, including the relative proportions of α-helices, extended strands, β-turns, and random coils, were predicted using the SOPMA online server (Geourjon and Deleage, 1995; https://npsa-prabi.ibcp.fr/cgi-bin/npsa_automat.pl?page=/NPSA/npsa_sopma.html). All identified proteins were subsequently submitted to the SWISS-MODEL server (Waterhouse et al., 2018; https://swissmodel.expasy.org/) to generate three-dimensional models, where the best-fitting and highest-similarity homology templates were selected. The templates with high GMQE and sequence identity were then employed for structural visualization and model construction using PyMOL (DeLano, 2002; https://pymol.org/).

### Phylogenetic analysis

The full-length amino acid sequences of 4CL proteins from *A. shenzhenica*, *D. catenatum*, *P. aphrodite*, *P. bellina*, *P. equestris*, *P. lueddemanniana*, *P. modesta* and *P. schilleriana* were aligned with their counterparts in *A. thaliana* (Li et al., 2015), *O. sativa* (Gui et al., 2011) and *Vanilla planifolia* (Kaur et al., 2025) using MUSCLE (Multiple Sequence Comparison by Log-Expectation) with gaps. Phylogenetic trees were then constructed using the neighbor-joining method in MEGAX (Kumar et al., 2018; http://www.megasoftware.net/) with pairwise deletion and 1000 bootstrap replicates.

### Gene structure and promoter analysis

The genomic and coding sequences (CDS) of *4CL* genes from *A. shenzhenica*, *D. catenatum*, and *P. equestris* were retrieved from the NCBI database. Gene structures were analyzed by comparing the genomic and coding sequences to determine the distribution of exons and introns using the Gene Structure Display Server (GSDS v2.0) (Hu et al., 2015; http://gsds.gao-lab.org/). Additionally, promoter sequences located 1.5 kb upstream of the start codon for these genes were obtained from NCBI. These promoter sequences were then analyzed using the PlantCARE database (Lescot et al., 2002; https://bioinformatics.psb.ugent.be/webtools/plantcare/html/) to predict the type and positions of *cis*-regulatory elements. The gene structure and promoter elements studies were only done for *A. shenzhenica*, *D. catenatum* and *P. equestris*, as genomic data were available for only these three out of the eight orchids under study.

### Expression profiling

The CDS (coding sequences) of genes were used in order to perform an NCBI Sequence Read Archive (SRA). BLASTn search was done against the RNA sequence data derived from the tissues of *A. shenzhenica* [pollen (SRX2938652), seed (SRX2938653), tuber (SRX2938654)], *D. catenatum* [root (SRX2938667), column (SRX2251512), sepal (SRX2251513), stem (SRX2251516), leaf (SRX2251517), lip (SRX2251518), flower bud (SRX2251519), pollinia (SRX2938662)], *P. aphrodite* [root (SRX2439763), leaf (SRX2439762), fully opened flower (SRX2439761), large flower bud (SRX2196321), small flower bud (SRX2439759)], *P. bellina* [leaf (SRX2210823), root (SRX2210822), large flower bud (SRX2210821), fully opened flower (SRX2210820)], *P. equestris* [sepal (SRX1806366), petal (SRX1806365), lip (SRX1806348), column (SRX1805894), flower bud (SRX1074880), leaf (SRX1074879), stem (SRX1074876), root (SRX1074875)], *P. lueddemanniana* [leaf (SRX2210814), root (SRX2210813), small bud (SRX2210812), large bud (SRX2210811), fully opened flower (SRX2210810)], *P. modesta* [small bud (SRX2210819), large bud (SRX2210818), fully opened flower (SRX2210817)] and *P. schilleriana* [large bud (SRX2210807), fully opened flower (SRX2210806)]. The number of hits were counted, and RPKM values were estimated as (C*10^9^)/(N*L), where *C* denotes the number of hits corresponding to a particular sequence, *N* denotes the total number of reads in that specific RNA-seq experiment and *L* denotes the length of the CDS sequence for the particular candidate gene (Mortazavi et al., 2008). Heatmaps were generated using the ClustVis tool (Metsalu and Vilo, 2015; https://biit.cs.ut.ee/clustvis/) to analyze the relative expression of all identified genes across different tissues in the selected orchid species.

## Results

**Figure 1:**
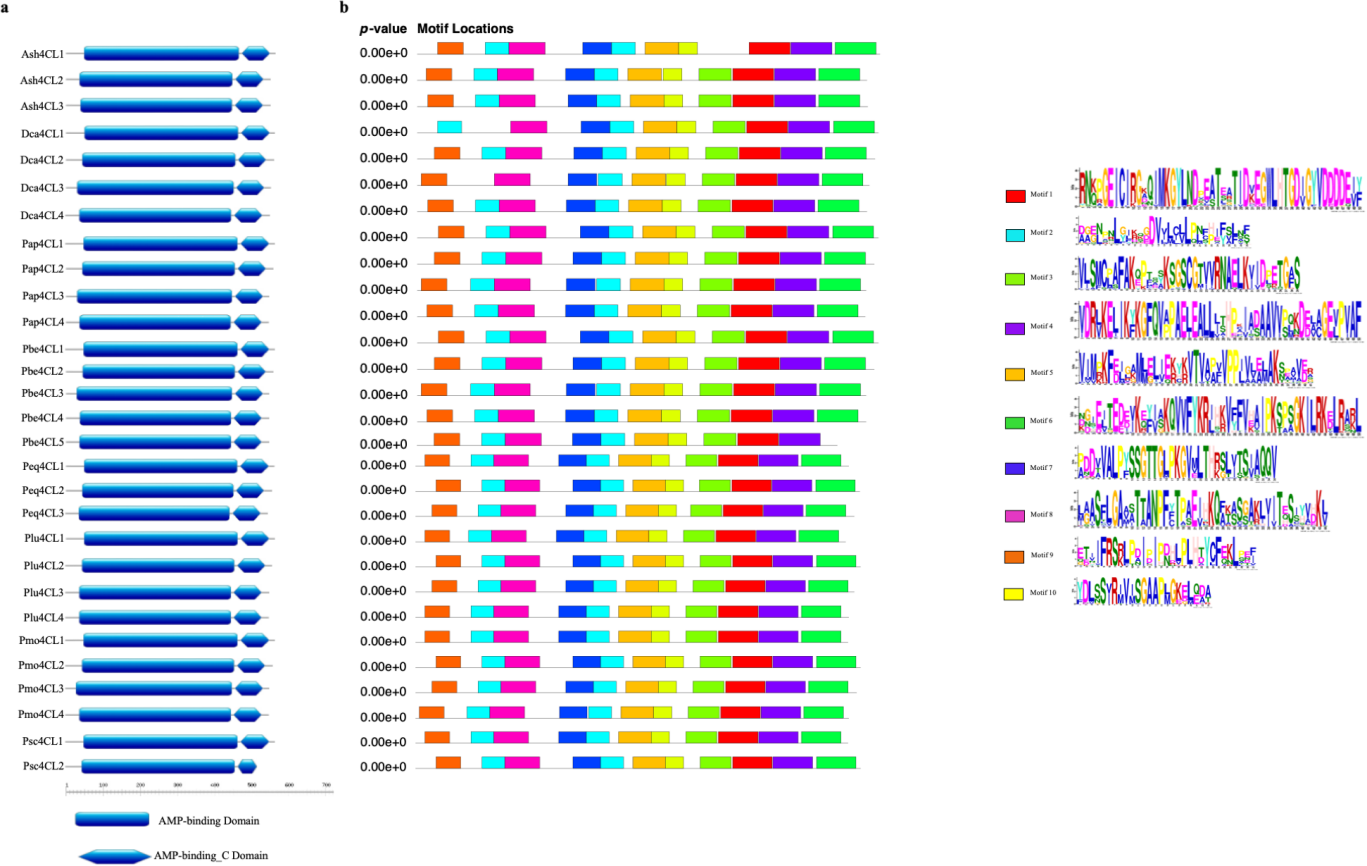
4CL proteins showing the position of **(a)** conserved domains and **(b)** conserved motifs.

Extensive BLASTp and tBLASTn searches led to the identification of 4CL proteins in selected orchid species using 4CL proteins of *Arabidopsis* and Rice as query sequences. The existence of two characteristic conserved domains, AMP-binding (PF00501) and AMP-binding_C (PF13193), was confirmed with the help of SMART server for validation of the predicted protein sequences. A total of three 4CL proteins in *A. shenzhenica* and *P. equestris* each, four in *D. catenatum*, *P. aphrodite, P. lueddemanniana* and *P. modesta* each, five in *P. bellina* and two in *P. schilleriana* were identified. Domain analysis and visualization to depict the presence of the conserved domain were done (**Supplementary Table 2**; **Figure 1**).

**Figure 2:**
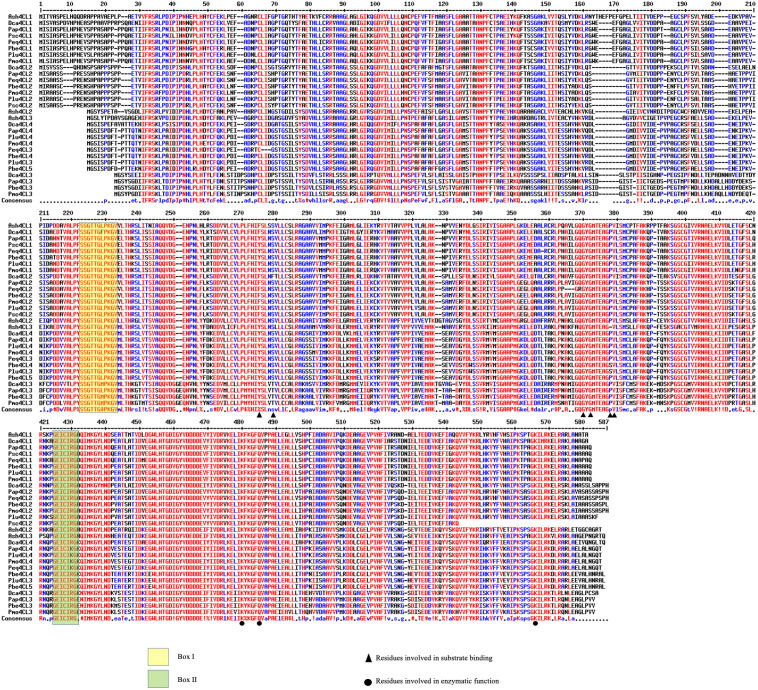
Multiple sequence alignment of 4CL proteins showing the presence of conserved regions marked by different colored boxes along with the residues involved in substrate binding and enzymatic function marked with black triangles and circles, respectively.

Upon aligning all the 4CL proteins, it was observed that the two distinct motifs, Box I (SSGTTGLPKGV) and Box II (GEICIRG), which are characteristic of 4CL proteins, were found conserved in all the proteins. Moreover, the conserved amino acid residues crucial for substrate binding and enzymatic function were also observed in the majority of the proteins (**Figure 2**).

In 4CL proteins, motifs 1 and 7 represented the AMP_binding domain and were consistently identified in all the proteins. Notably, motifs 7 and 1 contained the Box I (SSGTTGLPKGV) and Box II (GEICIRG) sequences, respectively, which are characteristic conserved regions of the 4CL proteins. Motif 4, present across all proteins, represented the AMP binding_C domain and contained the essential residues required for the enzymatic function of 4CLs (**Figure 1**).

Physicochemical characterization of 4CL proteins was done. The average length of these proteins was found to be 553 amino acids (aa). The smallest predicted protein was Psc4CL2 with a length of 514 aa, while the longest was Ash4CL1 with a length of 565 aa. The average molecular weight was predicted to be 59.97 kDa. Notably, Ash4CL1 exhibited the highest molecular weight (61.27 kDa) among all the 4CL proteins. The isoelectric point across all 4CL proteins ranged from 5.25 (Psc4CL2) to 6.6 (Peq4CL2), with an average of 5.86. Interestingly, all the proteins exhibited a positive GRAVY value except Pbe4CL5. Seventeen proteins were found to be stable based on protein stability analysis. The aliphatic index of the identified 4CL proteins ranged from 94.98 to 106.45, with the majority of proteins exhibiting values above 95, indicating the high abundance of aliphatic amino acids in these proteins. All 4CL proteins were localized in the peroxisome (**Table 2**).

**Table 2:** Physicochemical parameters of 4CL proteins.

Protein	AA	MW (kDa)	pI	Instability index	Aliphatic index	GRAVY	Subcellular localization
Ash4CL1	565	61.27	5.5	39.01	99.24	0.132	Peroxisome
Ash4CL2	549	59.55	5.78	37.3	101.73	0.087	Peroxisome
Ash4CL3	550	60.01	5.46	39.51	97.53	0.05	Peroxisome
Dca4CL1	563	60.53	5.4	39.93	100.85	0.194	Peroxisome
Dca4CL2	559	60.41	5.78	45.16	97.87	0.113	Peroxisome
Dca4CL3	552	60.40	5.9	42.19	96.9	0.043	Peroxisome
Dca4CL4	549	59.76	5.43	37.13	102.66	0.09	Peroxisome
Pap4CL1	563	60.64	5.59	40.06	104.49	0.198	Peroxisome
Pap4CL2	558	59.90	6.32	41.13	103.98	0.23	Peroxisome
Pap4CL3	548	59.90	5.99	40.5	96.42	0.009	Peroxisome
Pap4CL4	547	59.63	6.04	37.81	99.27	0.023	Peroxisome
Pbe4CL1	563	60.53	5.83	39.56	102.61	0.193	Peroxisome
Pbe4CL2	558	60.10	6.26	40.81	106.45	0.27	Peroxisome
Pbe4CL3	548	60.05	6.09	43.38	94.98	0.005	Peroxisome
Pbe4CL4	548	60.29	5.87	39.73	100.31	0.013	Peroxisome
Pbe4CL5	548	60.28	5.8	40.64	99.62	-0.006	Peroxisome
Peq4CL1	562	60.57	5.76	39.3	102.78	0.183	Peroxisome
Peq4CL2	555	59.60	6.6	42.13	101.89	0.18	Peroxisome
Peq4CL3	544	59.27	6.04	38.07	98.38	0.022	Peroxisome
Plu4CL1	563	60.62	5.64	38.58	106.06	0.226	Peroxisome
Plu4CL2	555	59.83	6.47	40.77	104.4	0.239	Peroxisome
Plu4CL3	548	60.27	5.88	39.01	101.02	0.01	Peroxisome
Plu4CL4	547	59.65	6.14	38.12	99.8	0.029	Peroxisome
Pmo4CL1	563	60.59	5.74	38.37	104.16	0.199	Peroxisome
Pmo4CL2	558	60.09	6.53	42.14	105.93	0.261	Peroxisome
Pmo4CL3	548	60.07	6.29	41.51	97.66	0.029	Peroxisome
Pmo4CL4	547	59.72	6.04	37.39	99.09	0.029	Peroxisome
Psc4CL1	563	60.70	5.57	39.51	103.62	0.19	Peroxisome
Psc4CL2	514	55.02	5.25	39.69	104.53	0.256	Peroxisome

AA, Amino acid; MW, Molecular weight (in kDa); pI, Theoretical isoelectric point; GRAVY, Grand average of hydropathicity index.

**Figure 3:**
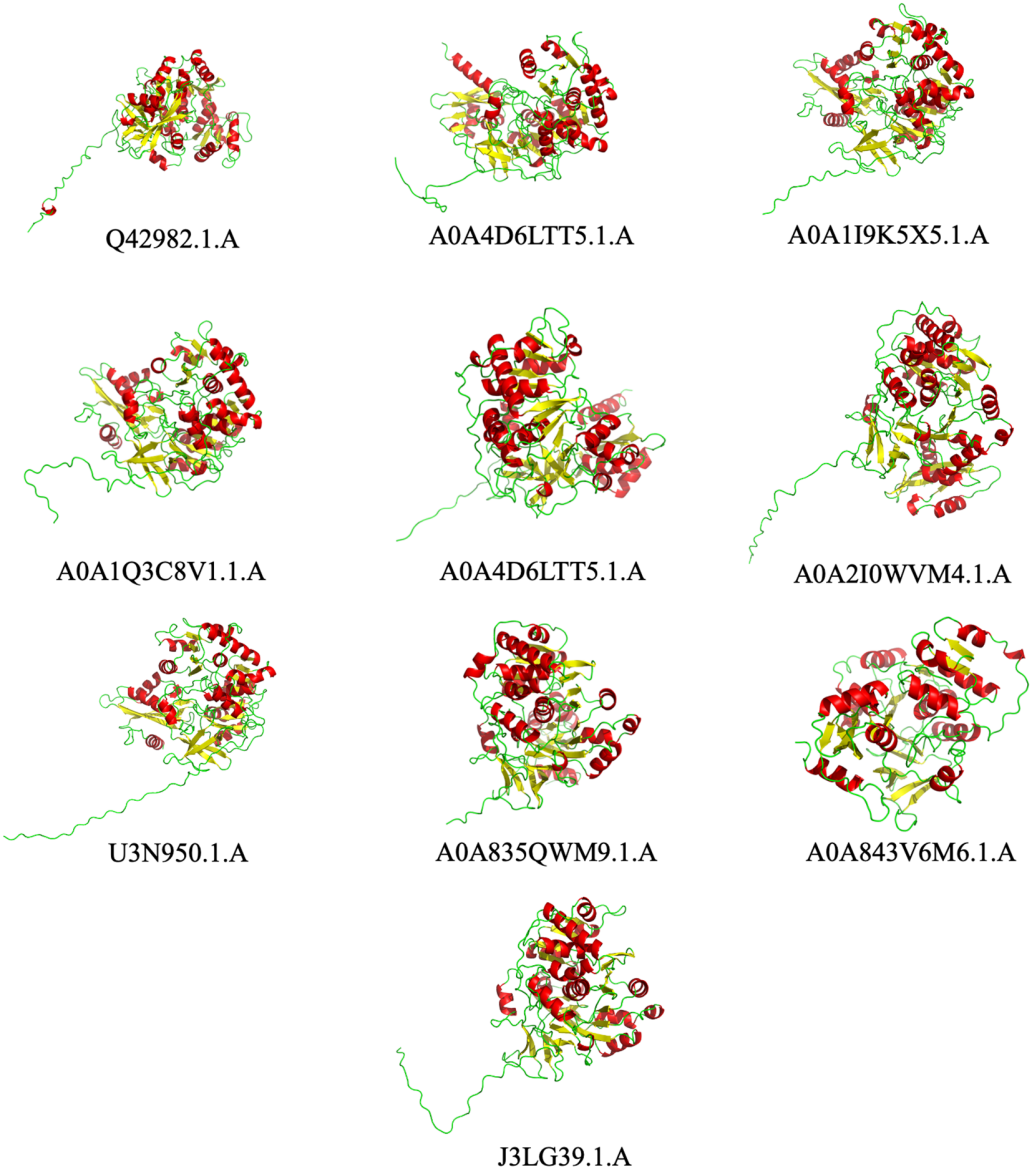
Three-dimensional model of selected templates of 4CL proteins.

The comparative analysis revealed a high degree of similarity in the predicted secondary structure of 4CL proteins. Interestingly, in 4CL proteins, random coils constituted the highest percentage compared to other secondary structure elements. The prevalence of random coils ranged from 37.23% in Pbe4CL4 and Pbe4CL5 to 43.37% in Pmo4CL2 (**Table 3**; **Supplementary Figure 1**). The prediction of tertiary structures of the identified 4CL proteins showed that the proteins showed homology to various PDB structure templates. However, all the templates represented 4-coumarate: CoA ligase proteins (**Supplementary Table 3**). The three-dimensional visualization of 4CL proteins based on the most commonly occurring and high-similarity templates was done using PyMOL (**Figure 3**).

**Table 3:** Prediction of Secondary structures of 4CL proteins.

Sequence ID	Alpha helix	Random coil	Extended strand	Beta turn
Ash4CL1	181 (32.04%)	233 (41.24%)	109 (19.29%)	42 (7.43%)
Ash4CL2	167 (30.42%)	223 (40.62%)	113 (20.58%)	46 (8.38%)
Ash4CL3	168 (30.55%)	226 (41.09%)	114 (20.73%)	42 (7.64%)
Dca4CL1	163 (28.95%)	235 (41.74%)	118 (20.96%)	47 (8.35%)
Dca4CL2	166 (29.70%)	240 (42.93%)	109 (19.50%)	44 (7.87%)
Dca4CL3	172 (31.16%)	222 (40.22%)	112 (20.29%)	46 (8.33%)
Dca4CL4	170 (30.97%)	232 (42.26%)	104 (18.94%)	43 (7.83%)
Pap4CL1	170 (30.20%)	233 (41.39%)	116 (20.60%)	44 (7.82%)
Pap4CL2	168 (30.11%)	236 (42.29%)	116 (20.79%)	38 (6.81%)
Pap4CL3	168 (30.66%)	221 (40.33%)	117 (21.35%)	42 (7.66%)
Pap4CL4	180 (32.91%)	217 (39.67%)	110 (20.11%)	40 (7.31%)
Pbe4CL1	176 (31.26%)	235 (41.74%)	111 (19.72%)	41 (7.28%)
Pbe4CL2	172 (30.82%)	239 (42.83%)	108 (19.35%)	39 (6.99%)
Pbe4CL3	166 (30.29%)	225 (41.06%)	112 (20.44%)	45 (8.21%)
Pbe4CL4	192 (35.04%)	204 (37.23%)	109 (19.89%)	43 (7.85%)
Pbe4CL5	193 (35.22%)	204 (37.23%)	107 (19.53%)	44 (8.03%)
Peq4CL1	172 (30.60%)	232 (41.28%)	110 (19.57%)	48 (8.54%)
Peq4CL2	161 (29.01%)	238 (42.88%)	112 (20.18%)	44 (7.93%)
Peq4CL3	181 (33.27%)	211 (38.79%)	109 (20.04%)	43 (7.90%)
Plu4CL1	177 (31.44%)	232 (41.21%)	113 (20.07%)	41 (7.28%)
Plu4CL2	176 (31.71%)	232 (41.80%)	109 (19.64%)	38 (6.85%)
Plu4CL3	190 (34.67%)	199 (36.31%)	116 (21.17%)	43 (7.85%)
Plu4CL4	183 (33.46%)	217 (39.67%)	108 (19.74%)	39 (7.13%)
Pmo4CL1	176 (31.26%)	234 (41.56%)	109 (19.36%)	44 (7.82%)
Pmo4CL2	162 (29.03%)	242 (43.37%)	114 (20.43%)	40 (7.17%)
Pmo4CL3	172 (31.39%)	226 (41.24%)	107 (19.53%)	43 (7.85%)
Pmo4CL4	181 (33.09%)	212 (38.76%)	110 (20.11%)	44 (8.04%)
Psc4CL1	177 (31.44%)	235 (41.74%)	114 (20.25%)	37 (6.57%)
Psc4CL2	147 (28.60%)	216 (42.02%)	117 (22.76%)	34 (6.61%)

**Figure 4:**
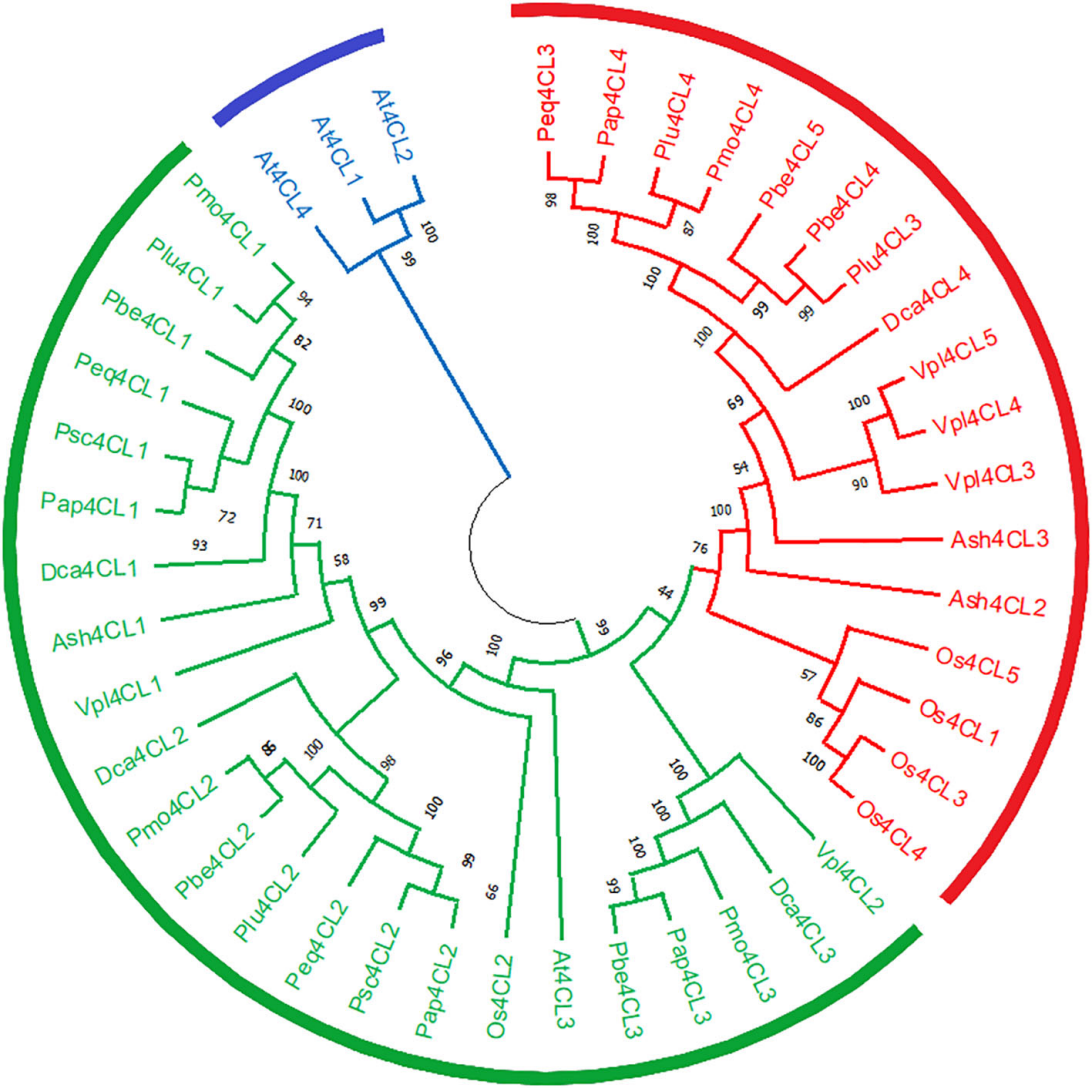
Phylogenetic analysis of 4CL protein sequences, wherein blue, green and red colors represent the class I, class II and class III of 4CL proteins, respectively.

MEGA software was employed to explore the evolutionary connections among the 4CL protein sequences of *A. schenzhenica, D. catenatum, P. aphrodite, P. bellina, P. equestris, P. lueddemanniana, P. modesta*, *P. schilleriana*, *V. planifolia*, *A. thaliana* and *O. sativa*. The phylogenetic analysis of 4CL proteins revealed a tight clustering of Ash4CL2, Ash4CL3, Dca4CL4, Pap4CL4, Pbe4CL4-5, Pe4CL3, Pmo4CL4, Plu4CL4, Vpl4CL3–5 with class III 4CL proteins of *O. sativa* (Os4CL1, Os4CL3, Os4CL4 and Os4CL5) while the remaining orchid proteins clustered with class II members of 4CL proteins in *Arabidopsis thaliana* (At4CL3) and *Oryza sativa* (Os4CL2). However, no orchid protein sequence clustered in class I of 4CL proteins comprising At4CL1, At4CL2 and At4CL4 (**Figure 4**).

**Figure 5:**
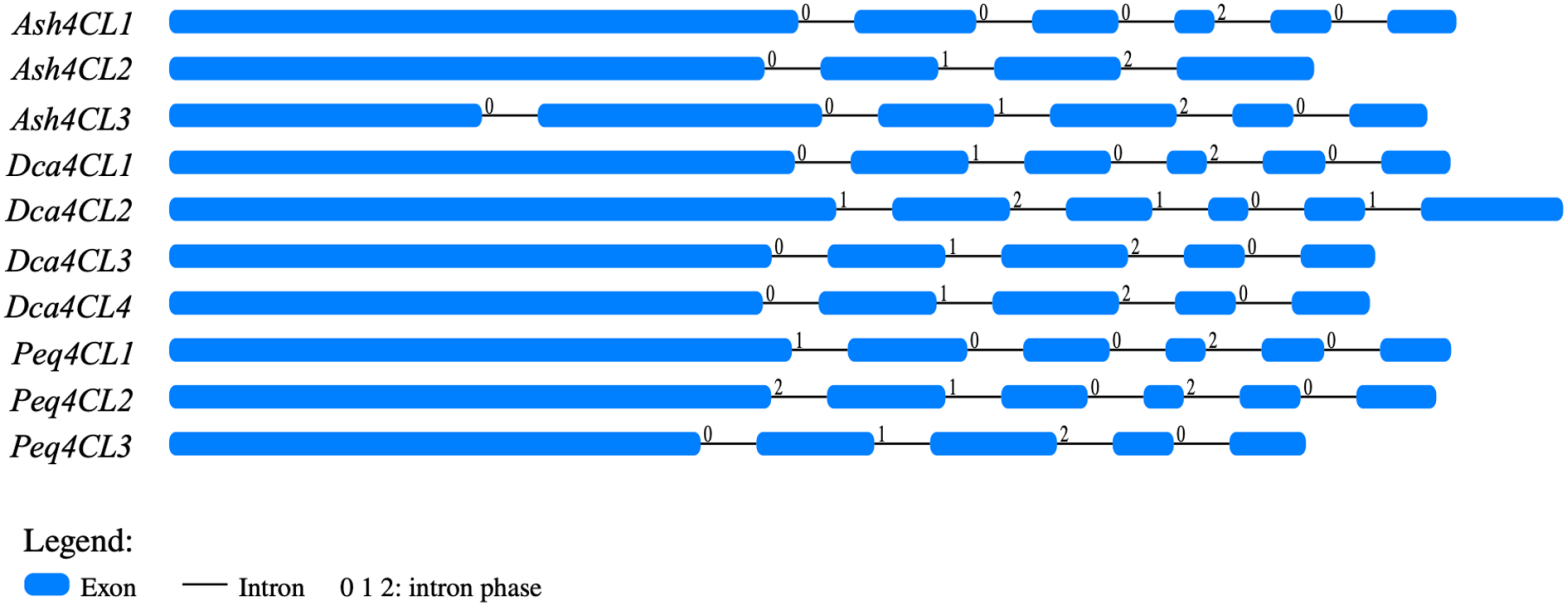
Gene structure of *4CL* genes.

The Gene Structure Display Server (GSDS) online tool was used for gene structural analysis. Notably, *4CL* genes exhibited a diverse intron-exon structure. *Dca4CL3–4* and *Peq4CL3* genes featured four introns and five exons, while *Ash4CL1*, *Ash4CL3*, *Dca4CL1–2* and *Peq4CL1–2* displayed five introns and six exons and *Ash4CL2* exhibited three introns and four exons (**Figure 5**). Comprehensive details regarding the gene scaffold and gene stretch region of *4CL* genes are provided in **Table 4**.

**Table 4:** Characterization of *Ash4CL*, *Dca4CL* and *Peq4CL* genes.

Gene	Location in genome	Gene stretch	Number of exons	Number of introns
*Ash4CL1*	Loci: fragScaff_scaffold_95	c1187152-1176385	6	5
*Ash4CL2*	Loci: fragScaff_scaffold_80	c433741-426298	4	3
*Ash4CL3*	Loci: fragScaff_scaffold_155	c1160315-1152316	6	5
*Dca4CL1*	Loci: fragScaff_scaffold_716	20371336-20376294	6	5
*Dca4CL2*	Loci: fragScaff_scaffold_601	c227168-221460	6	5
*Dca4CL3*	Loci: fragScaff_scaffold_1126	c695704-693488	5	4
*Dca4CL4*	Loci: fragScaff_scaffold_621	c11330335-11325764	5	4
*Peq4CL1*	Loci: Scaffold000369	c774561-766809	6	5
*Peq4CL2*	Loci: Scaffold000202	1738006-1751433	6	5
*Peq4CL3*	Loci: Scaffold000002	14068300-14073361	5	4

*c in the gene stretch column in the above table stands for complementary strand.

**Figure 6:**
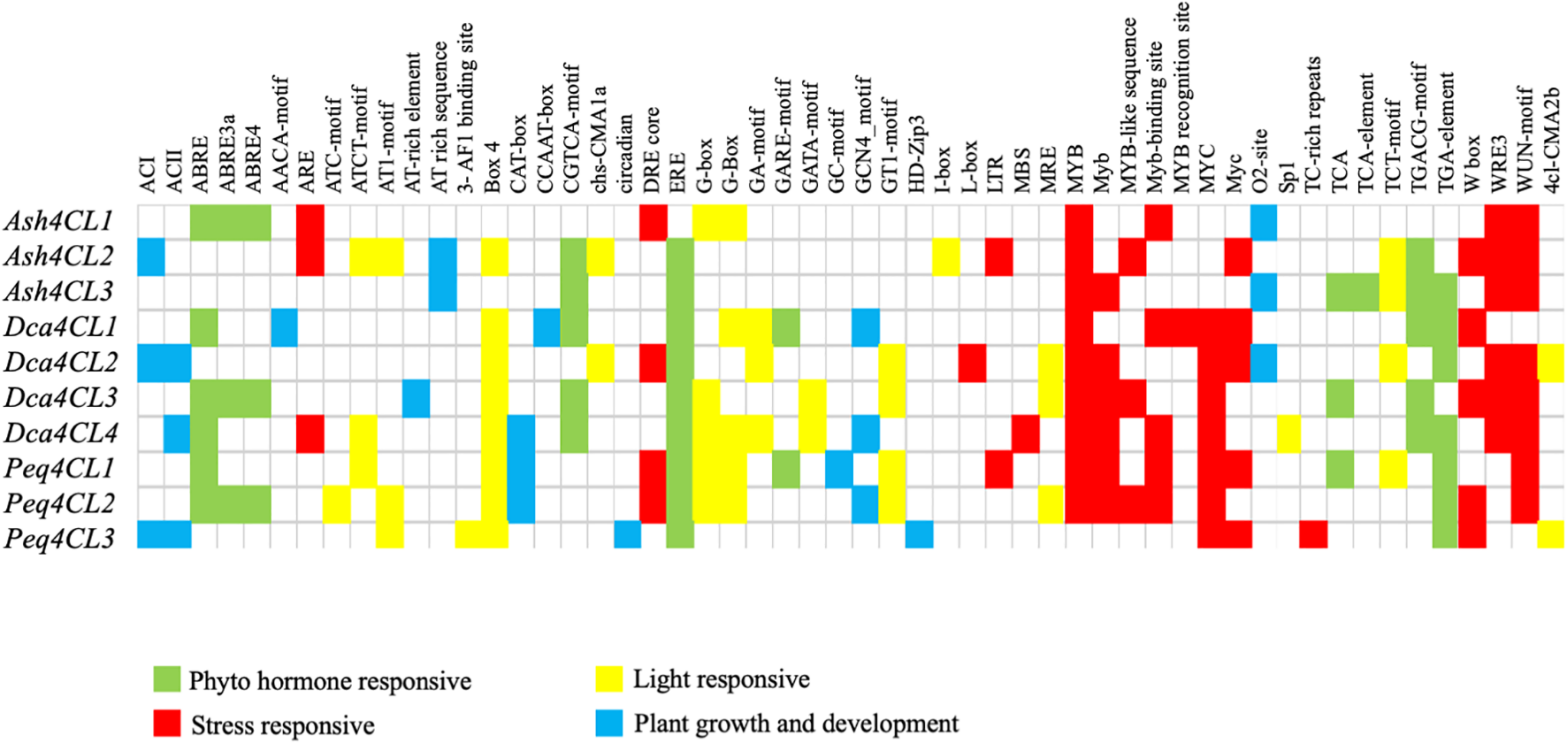
Various *cis*-regulatory elements present in the promoter region of *4CL* genes. Phytohormone-responsive, light-responsive, abiotic and biotic stress-responsive and growth and development-regulating elements are represented by green, yellow, red and blue color, respectively.

In the examined promoter sequences of *4CL* genes, besides the commonly found promoter elements such as TATA and CAAT boxes, our investigation revealed the presence of 54 other elements. These elements play pivotal roles in regulating four fundamental plant responses like plant growth and development, phytohormone response, abiotic & biotic stress response and light response. Notably, stress-responsive, phytohormone-responsive and light-responsive elements were found in greater abundance compared to elements governing plant growth and development (**Supplementary Table 4**). Crucial *cis*-acting elements identified in this study, such as ACI, ACII, O2-site, CAT-box, CCAAT box and GCN4_motif, were implicated in influencing plant growth and development. Elements demonstrating phytohormone responsiveness included ABRE, CGTCA-motif, ERE, GARE-motif, TGACG-motif, and TCA-element. These elements can be induced by different hormones such as abscisic acid, gibberellins, salicylic acid and methyljasmonate. Stress-responsive elements detected encompassed ARE, DRE core, LTR, MYB, MYC, MYB-like sequence, W-Box, WRE3, and WUN-motif. Additionally, various light-responsive *cis*-acting elements, such as Box 4, G-Box, GA motif, GATA motif, GT1-motif, MRE and chs-CMA1a were also identified (**Figure 6**).

**Figure 7:**
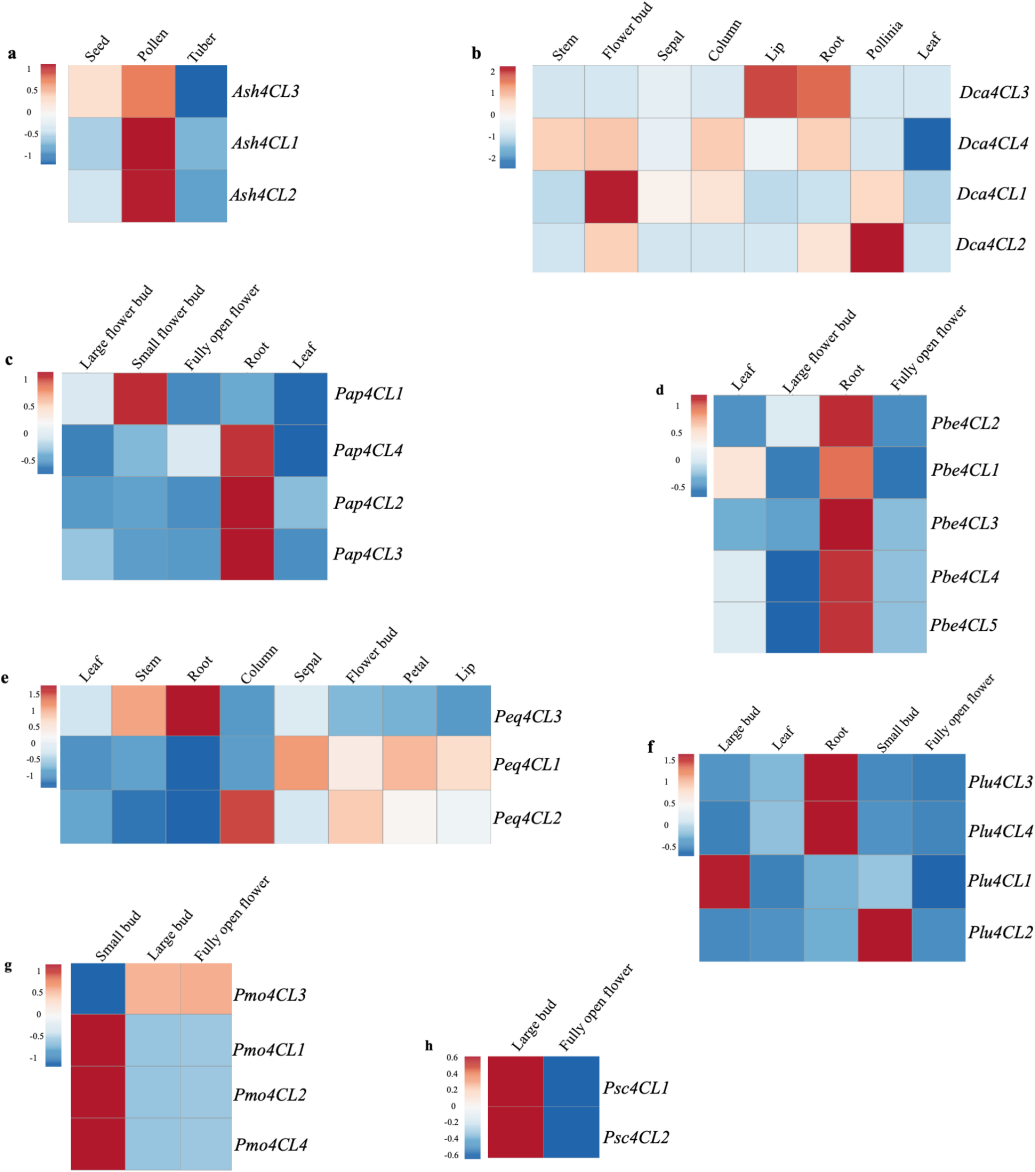
Expression profile of 4CL genes: **(A)** Apostasia shenzhenica, **(B)** Dendrobium catenatum, **(C)** Phalaenopsis aphrodite, **(D)** Phalaenopsis bellina, **(E)** Phalaenopsis equestris, **(F)** Phalaenopsis lueddemanniana, **(G)** Phalaenopsis modesta, **(H)** Phalaenopsis schilleriana.

Expression analysis played a pivotal role in discerning the variations in *4CL* genes expression across diverse vegetative and reproductive tissues of orchids under study. *Ash4CL1* and *Ash4CL2* were expressed highly in pollen in comparison to the seed and tuber. *Dca4CL2* of *Dendrobium catenatum* also showed high expression in pollinia. *Pbe4CL1–5* of *Phalaenopsis bellina*, *Pap4CL2–4* of *Phalaenopsis aphrodite*, *Peq4CL3* of *Phalaenopsis equestris* and *Plu4CL3–4* of *Phalaenopsis lueddemanniana* were highly expressed in the root compared to other tissues. *Pmo4CL1*, *Pmo4CL2* and *Pmo4CL4* showed maximum expression in the small bud, while *Psc4CL1* and *Psc4CL2* displayed elevated expression in the large bud (**Supplementary Table 5**; **Figure 7**).

## Discussion

The rich diversity of secondary metabolites in orchids underlies their remarkable curative potential, highlighting the importance of investigating their biosynthetic pathways. Phenolics are a large and important class of secondary metabolites, and the pathway involved in the biosynthesis of phenolic compounds is the phenylpropanoid pathway. The current research was aimed at identification and characterization of 4 CL, one of the key enzymes operating at a terminal stage of the phenylpropanoid metabolic pathway and contributing to the synthesis of phenylpropanoids, which act as precursor molecules for the production of a wide range of compounds involved in plant growth and development, defense against pathogens, and response to environmental cues (Ferrer et al., 2008; Vogt, 2010). The number of *4CL* genes identified across various plant species has been quite variable. The prediction of five *4CL* genes in *P. bellina* corresponds to the reports in *O. sativa* (Gui et al., 2011) and *Populus tomentosa* (Rao et al., 2015), where both of these plants also exhibited five *4CL* genes. The *4CL* gene family in *D. catenatum, P. aphrodite, P. modesta* and *P. lueddemanniana* comprises four members, which is comparable to the presence of the *4CL* gene family in the model plant *A. thaliana* (Li et al., 2015). Three *4CL* genes have been identified in *A. shenzhenica* and *P. equestris* in the present work. Other plants which show such reduced numbers include *Rubus idaeus* (Kumar and Ellis, 2003), *Boehmeria nivea* (Tang et al., 2018), *Piper nigrum* (Jin et al., 2020), and *Citrus sinensis* (Zhou et al., 2022). Similarity in the quantitative strength of *4CL* genes in *P. schilleriana* (two *4CL* genes) is observed with *Panicum virgatum* (Xu et al., 2011) and *Salvia miltiorrhiza* (Jin et al., 2012). However, there are plant species in which the number of *4CL* genes is quite high, which may be due to evolutionary differences caused by gene duplication events.

Further, protein domain and motif studies shed insights into numerous conserved characteristics of the proteins. In 4CL proteins, two domains were predicted: AMP-binding and AMP-binding_C. The two signature motifs of the 4CL proteins, Box I (SSGTTGLPKGV) and Box II (GEICIRG), are present in 4CL proteins in all plants, and sequence alignment of the orchid 4CL proteins also showed the same conservation, thus it is consistent with the studies in other plants (Uhlmann and Ebel, 1993; Lee et al., 1995; Wei et al., 2013; Gao et al., 2015; Zhang et al., 2019; Sun et al., 2020; Wang et al., 2022, 2023). This highlights the evolutionary stability of 4CL proteins across various plant species. All proteins in the family of adenylate-forming enzymes have Box I, which is the AMP (adenosine monophosphate) nucleotide binding motif (Allina et al., 1998; Lavhale et al., 2018). Furthermore, according to Stuible et al. (2000), the cysteine in the GEICIRG motif contributes to the stability and catalytic activity of 4CLs. Further, identical motif organization was detected in 4CL proteins of orchids compared to *Arabidopsis thaliana* and *Oryza sativa* 4CLs, which corroborated that the gene family is very conserved and the proteins are likely to perform a similar role in the phenylpropanoid pathway.

The characteristics of the inferred proteins were described using various bioinformatics methods. 4CL proteins were found in the peroxisome, resembling Pg4CL10 of *Punica granatum*, numerous 4CL members of *Gossypium hirsutum* (Sun et al., 2020), Ml4CL1–10 of *Miscanthus lutarioriparius* (Huang et al., 2026) and Le4CL3 and Le4CL4 of *Lithospermum erythrorhizon* (Nakanishi et al., 2024). All of the 4CL proteins, except Pbe4CL5 had positive GRAVY values, which is consistent with a prior study on the hydrophobicity of Bn4CL3 from *Boehmeria nivea* (Tang et al., 2018), Pg4CL1-3, Pg4CL6, and Pg4CL8–11 from *Punica granatum* (Wang et al., 2022), Ma4CLs of *Musa acuminata* (Wang et al., 2024), Vpl4CL1–3 and Vpl4CL5 of *Vanilla planifolia* (Kaur et al., 2025) and Ml4CLs of *Miscanthus lutarioriparius* (Huang et al., 2026). Further, twelve 4CL proteins under study exhibited an instability index of more than 40, which indicates that these proteins may require post-translational modifications for their functioning (Lee et al., 2023). Additionally, the high aliphatic index for the majority of proteins indicated their high thermostability (Ikai, 1980). Molecular modelling techniques differentiated the various proteins under study on the basis of the amount of beta turns, extended strands, random coils, and alpha helices. In addition, 3-D structure analysis showed the resemblance of the proteins to 4-coumarate: CoA ligase templates based on homology analysis.

In the case of 4CL proteins identified in the present study in orchid species, the proteins belonging to classes I, II, and III formed different clades. In dicots, 4CL proteins are divided into 2 classes, with Class I proteins playing a role in lignin biosynthesis while the Class II proteins are involved in the biosynthesis of other phenolic compounds (Ehlting et al., 1999). But in the case of *Oryza sativa*, a monocot, the proteins are clustered into 3 clades, Class I, II and III, similar to our present results. Thus, this indicates that there are variations in substrate specificity of 4CL enzymes in monocots and dicots, eventually leading to different compositions of compounds produced amongst these groups of plants. Furthermore, a close grouping of specific proteins in the phylogenetic tree illustrated their strong evolutionary links.

Gene architecture studies were performed by evaluating the number of exons/introns as well as intronic phases. 60% of the orchid *4CL* genes under study exhibited five introns and 6 exons, similar to *Ma4CL1-1b*, *Ma4CL2*, *Ma4CL3-2*, *Ma4CL5-1*, *Ma4CL5-2*, *Ma4CL8-1*, *Ma4CL11-1*, and *Ma4CL11–3* of *Musa acuminata* (Wang et al., 2024) and *Ml4CL7* and *Ml4CL8* of *Miscanthus lutarioriparius* (Huang et al., 2026). *4CL* genes identified in the present work were found to have a variety of phytohormone-responsive, abiotic and biotic stress-responsive, inducers of plant growth and development, and light-responsive *cis*-elements in their promoter regions. Similar *cis*-acting elements of 4 categories were predicted for the *Punica granatum* (Wang et al., 2022), *Triticum aestivum* (Gholizadeh et al., 2025), *Gastrodia elata* (Sha et al., 2025) and *Zea mays* (Zhang Z. et al., 2025a) *4CL* genes. It has also been shown that the *4CL* genes of *Gossypium hirsutum* (Sun et al., 2020), *Eucommia ulmoides* (Zhong et al., 2022) and *Nicotiana tabacum* (Ma et al., 2026) contain *cis*-acting elements that regulate stress and phytohormone responses. The presence of several *cis*-regulatory elements related to phytohormones and stress responses, including MYB and MYC binding sites, suggests that *4CL* genes may be involved in plant responses to various biotic and abiotic stresses. MYB and MYC transcription factors are known to play important roles in regulating stress responses in plants, supporting the possible involvement of *4CL* genes in plant resilience under changing environmental conditions (Liu et al., 2024).

The expression profiles of *4CL* genes varied depending on the different vegetative and reproductive organs. *Ash4CL1*, *Ash4CL2* and *Dca4CL2* in the present study expressed highly in the male reproductive tissues, similar to the high expression of *Os4CL2* in anthers of *O. sativa* (Sun et al., 2013) and *Zm4CL27* in anthers of *Zea mays* (Zhang Z. et al., 2025a). This indicates their potential function in male tissue development. Roots showed increased expression of *Pbe4CL1–5* of *P. bellina*, *Pap4CL2–4* of *P. aphrodite*, *Peq4CL3* of *P. equestris* and *Plu4CL3–4* of *P. lueddemanniana*. *At4CL1* and *At4CL2* of *A. thaliana* (Lee et al., 1995), *Pto4CL1*, *Pto4CL4*, and *Pto4CL5* of *Populus tomentosa* (Rao et al., 2015). *4CL* genes like *Ii4CL* of *Isatis indigotica* (Di et al., 2012), *Pg4CL5* of *Punica granatum* (Wang et al., 2022), *Ri4CL2–3* of *Rubus idaeus* (Kumar and Ellis, 2003), *Le4CL3* and *Le4CL4* of *Lithospermum erythrorhizon* (Nakanishi et al., 2024), *Nt4CL5*, *Nt4CL7*, and *Nt4CL8* of *Nicotiana tabacum* (Ma et al., 2026) and *Ta4CL90*, *Ta4CL96*, and *Ta4CL99* of *Triticum aestivum* (Zhang Z. et al., 2025b) all showed similar findings for high expression in the root which suggests the role of these genes in roots. Similarly, *Bna4CL1, Bna4CL3*, *Bna4CL4, Bna4CL8* and *Bna4CL11* of *Brassica napus* showed significantly higher expression in roots and stems (Zhang M. et al., 2025). This suggests that these genes are likely also involved in the vegetative growth of plants.

Further, more research on the regulation of the 4CL enzymes involved in the phenylpropanoid pathway can provide insights into plant metabolic processes and how they adapt to different environmental conditions. Additionally, by comprehending the mechanisms that underlie the activity of 4CLs, specific targeted genetic modifications may also be made to improve the abundance of desired substances in plants.

## Conclusions

In the present study, we identified and characterized 29 *4CL* genes in eight orchid species. The number and physicochemical properties of the identified proteins were found to be variable amongst the different orchid species under study. Phylogenetic analysis showed the clustering of *4CL* genes into three clades. Our results provided an understanding of the potential functions of *4CL* genes. However, the functional characterization and validation of the identified genes would be the part of future research.

## Data Availability

The original contributions presented in the study are included in the article/**Supplementary Material**. Further inquiries can be directed to the corresponding author.

## Supplementary material

Supplementary Figure 1 4CL proteins showing the distribution of secondary structures.
